# Social Isolation, Acculturative Stress, and Resilience Among Older Asian Indian Immigrants in the United States: A Qualitative Study

**DOI:** 10.1002/puh2.70365

**Published:** 2026-09-10

**Authors:** Malinee Neelamegam, Shilpa Patil, RoiSan Nhpang, Sarah Alkhatib, Anika Gupta, Nolan Kline, Stacey Griner

**Affiliations:** ^1^ Department of Population and Community Health College of Public Health University of North Texas Health Science Center Fort Worth Texas USA; ^2^ Texas College of Osteopathic Medicine University of North Texas Health Science Center Fort Worth Texas USA; ^3^ Department of Population Health Sciences College of Medicine University of Central Florida Orlando Florida USA

**Keywords:** aging, loneliness, older immigrant, social isolation, South Asian

## Abstract

**Background:**

Social isolation and loneliness are increasingly recognized as public health concerns for aging populations, yet the experiences of older Asian Indian immigrants in the United States (US) remain understudied. This qualitative study explored how immigration, acculturation, and changes in social networks shape isolation, loneliness, and resilience among older Asian Indian immigrants.

**Methods:**

In‐depth interviews were conducted with 29 Asian Indian immigrants aged 50 years and older in the Dallas–Fort Worth metroplex. Participants were recruited through purposive sampling with support from local cultural and faith‐based organizations. A semi‐structured interview guide examined social networks, isolation, loneliness, acculturation, cultural identity, and coping. Interviews were audio‐recorded, transcribed verbatim, translated when necessary, and analyzed using inductive thematic analysis. Descriptive statistics summarized participant characteristics.

**Results:**

Participants described substantial disruption of social networks after immigration, including fewer close friendships, reduced spontaneous social interactions, and limited nearby family support. These changes contributed to loneliness and emotional strain, particularly in an individualistic social environment that contrasted with communal norms in India. Participants also described acculturative stress related to preserving Indian cultural identity while adapting to US society, especially in intergenerational family contexts. Despite these challenges, many used resilience strategies such as staying active, lifelong learning, maintaining optimism, and seeking supportive social circles.

**Conclusion:**

Social isolation among older Asian Indian immigrants reflects a public health challenge shaped by migration, cultural adaptation, family dispersion, and limited culturally relevant supports. Community‐based, culturally tailored interventions are needed to strengthen social connection, reduce loneliness, and promote healthy aging in immigrant communities.

## Graphical abstract

Older Asian Indian immigrants in the United States described disrupted social networks, loneliness, and acculturative stress tied to preserving cultural identity, alongside resilience through activity, optimism, and supportive social circles. Culturally tailored, community‐based interventions are needed to strengthen social connection and support healthy aging.

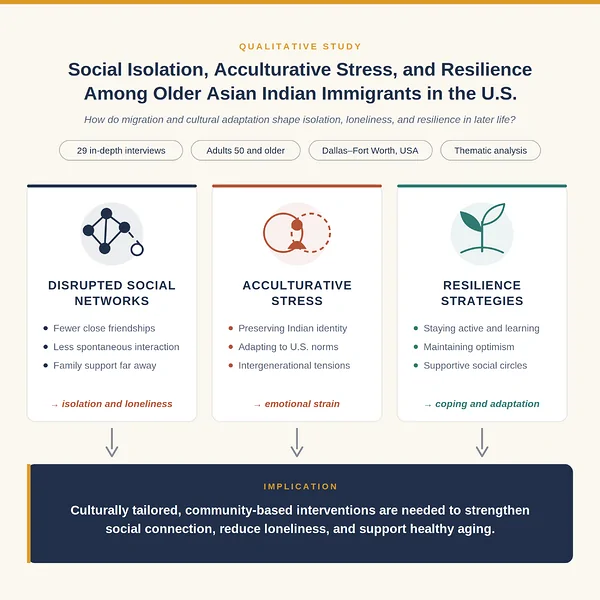

## 1 Introduction

The Asian Indian population in the United States (US) continues to grow, making it the second‐largest immigrant group in the country [1]. This population in the US saw a 142% population growth in the last two decades [2]. While often perceived as a predominantly young community, approximately 20% of Asian Indians are aged 50 and older, with the average age being 40 years [1, 2]. As the population ages, the number of older Asian Indians is expected to increase significantly. This demographic includes two distinct groups: long‐term residents who immigrated as professionals decades ago and older adults who immigrated later in life to reunite with family [3]. These groups differ substantially in demographic and socioeconomic profiles, with the latter group facing pronounced health disparities. For example, nearly 45% of older Asian Indian adults have less than a high school education, and 36% are not fluent in English, compounding the challenges of aging in a foreign country [4].

Social isolation, defined as the lack of meaningful social interactions and relationships, is a common experience among immigrant populations [5]. Importantly, social isolation is conceptually distinct from loneliness. Social isolation refers to an objective deficit in the number and frequency of social contacts, whereas loneliness is the subjective, distressing perception that one's social relationships are inadequate in quantity or quality [6, 7]. The two constructs do not always co‐occur, and each has been independently linked to adverse health outcomes in older adults [8]. In this study, we therefore attend both to objective disruptions of participants’ social networks (social isolation) and to their subjective experiences of disconnection (loneliness). This phenomenon is particularly relevant for Asian Indian immigrants, who often face challenges in forming and maintaining social connections in their new environment. Additionally, the process of acculturation, which involves cultural changes resulting from exposure to culturally dissimilar people, groups, and social influences, compounds these challenges [9]. Acculturation can lead to acculturative stress, a form of psychological stress associated with navigating the complexities of adapting to a new culture while maintaining one's cultural identity. This stress can manifest through feelings of marginalization, discrimination, and cultural dissonance [10]. For Asian Indians in the US, this issue is particularly pertinent, as the majority of the population were born and raised outside of the country [11]. This demographic is at heightened risk for these psychosocial stressors, as they often face barriers such as language differences, cultural disconnects, and limited access to culturally relevant resources. These factors not only exacerbate feelings of isolation but also have the potential to negatively impact mental health and overall well‐being, making it critical to address these psychosocial risk factors in this growing population.

Social isolation and acculturative stress are critical factors that significantly influence health outcomes among Asian Indians. Research highlights that social engagements and relationships play a vital role in shaping the well‐being and health behaviors of this population [12]. Psychosocial factors, including social isolation and acculturation stress, have been linked to poor health outcomes across a range of diseases in Asian Indians. For example, studies indicate that acculturation style impacts health, with individuals who adopt assimilation (a preference for US culture) or integration (a balanced preference for both Indian and US cultures) displaying better cardiometabolic profiles compared to those with a separation acculturation style (a strong preference for Indian culture) [13]. These findings underscore the importance of addressing psychosocial influences to improve health outcomes in this community. Despite their growing numbers, this population has been historically underrepresented in aging research. The purpose of our study is to examine the extent to which older Asian Indian immigrants in the US are impacted by social isolation, acculturative stress, and loneliness.

## 2 Theoretical Framework

This study is grounded in two theoretical concepts: Social Network Theory and Acculturation Theory, which provide critical perspectives on the experiences of older Asian Indian immigrants in the US. The social network theoretical perspective [14, 15] examines the structure and function of social relationships, highlighting the importance of bonding, bridging, and linking ties in influencing well‐being. It guided our exploration of how shifts in social networks post‐immigration contributed to loneliness and isolation. The acculturation theoretical perspective [16] explores how immigrants adapt to new cultural environments while maintaining their original identity, offering insights into the dual pressures of cultural assimilation and heritage preservation. These frameworks informed the study design, including the development of the interview guide and thematic analysis. Beyond informing the study design, these frameworks also guided our interpretation of the findings. We draw on the concepts of bonding, bridging, and linking ties to interpret participants’ changing social networks, and on Berry's acculturation strategies (integration, assimilation, separation, and marginalization) to interpret participants’ experiences of acculturative stress and cultural identity negotiation.

## 3 Methods

### 3.1 Setting

This study was conducted in Texas, home to the second‐largest Asian Indian population in the US, with the North Texas region witnessing substantial growth in this demographic over the last decade. The Dallas–Fort Worth (DFW) metroplex now ranks as the fourth‐largest metropolitan area in the US for Asian Indians [17]. The research received approval from the North Texas Regional Institutional Review Board (IRB).

### 3.2 Participants

The participants in this study were older Asian Indian immigrants living in the DFW metroplex. The eligibility criteria included: (1) being 50 years of age or older, (2) self‐identifying as Asian Indian, and (3) having immigrated to the US from India. To recruit participants, the research team utilized purposive sampling technique and collaborated with cultural and faith‐based organizations in the DFW area. Additionally, recruitment strategies from previous studies were utilized, such as distributing informational flyers through partner organizations, advertising on social media, active outreach at community events, and snowball sampling. In total, 29 eligible participants provided informed consent and completed in‐depth interviews.

### 3.3 Data Collection and Analysis

Before the in‐depth interviews, participants completed a brief demographic survey. A semi‐structured interview guide was used to explore participants’ lived experience related to social networks, isolation, and loneliness, related to their immigrant status (Table 1). The interviews were audio‐recorded, transcribed verbatim, and translated when necessary. Two team members conducted an inductive thematic analysis of the transcripts using MAXQDA software. The analysis followed the phases of thematic analysis described by Braun and Clarke [18]. The two coders first independently read and open‐coded an initial subset of transcripts to develop a preliminary codebook, which was iteratively refined through team discussion as coding proceeded. The remaining transcripts were then coded independently by both coders, and coding discrepancies were resolved through consensus discussion, with unresolved discrepancies adjudicated by the senior author. Analytic memos and an audit trail of coding decisions were maintained throughout the analysis. To support trustworthiness, we used strategies consistent with established criteria for credibility, dependability, and confirmability, including peer debriefing within the research team and reflexive discussion of team members’ positionality and assumptions in relation to the study population [19]. Member checking was not conducted, and we acknowledge this as a limitation. Additionally, descriptive analysis was carried out using SPSS software to generate summary statistics for the 29 participants. Frequencies and percentages were calculated for categorical variables, whereas means and standard deviations were reported for numerical variables. The study was approved by the North Texas Regional IRB.

**TABLE 1:** Interview questions.

1	Can you tell me a little about yourself? How long have you been in the United States?
2	What motivated you to move to the US, and what has your experience been like living here?
3	What was your social life like back in India?
	Probe: How does it compare to your social life in the US?
4	What has been your experience making friends or forming new relationships in the US?
5	Do you ever feel isolated or lonely? Can you share some specific experiences?
	Probe: What do you think is making you feel this way?
6	What challenges have you faced in building or maintaining relationships in the US?
7	How do cultural or language differences play a role in these challenges?
8	How have you coped with the challenges of living in a new country?
	Probe: Are there specific strategies or practices that have helped you stay positive and resilient?

Abbreviation: US, United States.

## 4 Results

The demographic characteristics of the study participants are presented in Table 2. The largest age groups were 55–64 years (34.5%) and 65–74 years (37.9%). The majority of participants were female (72.4%), and most were naturalized US citizens at the time of data collection (93.1%). On average, participants had been living in the US for 34.7 years (±12.7 years).

**TABLE 2:** Characteristics of the study participants.

Characteristics	Study sample (*n* = 29)
Age, *n* (%)	Frequency
50–54	3 (10.3)
55–64	10 (34.5)
65–74	11 (37.9)
75 and older	5 (17.2)
Gender, *n* (%)	
Man	8 (27.6)
Woman	21 (72.4)
Marital status, *n* (%)	
Married	25 (86.2)
Separated/Divorced	1 (3.4)
Widowed	3 (10.3)
Employment status, *n* (%)	
Currently employed	10 (34.5)
Never employed/homemaker	3 (10.3)
Previously employed/retired	16 (55.2)
Education level, *n* (%)	
No formal education	0 (0)
High school or less	0 (0)
College/Undergraduate	8 (27.6)
Graduate school	21 (72.4)
Current status in US, *n* (%)	
US citizen	27 (93.1)
Lawful permanent resident	2 (6.9)
Employment‐based visa	0 (0)
Other	0 (0)
Age at immigration, mean (SD)	31.86 (13.19)
Years since immigration, mean (SD)	34.66 (12.69)

Abbreviation: US, United States.

Our in‐depth interviews revealed several key themes related to the perspectives of older Asian Indian immigrants on social networks, isolation, and loneliness. These themes included (1) cultural adjustment and assimilation, (2) social isolation, loneliness, and the impact of immigration on social networks, (3) challenges in maintaining cultural identity, and (4) resilience and adaptation (Table 3).

**TABLE 3:** Summary of thematic analysis findings.

Theme	Respondent characteristics	Quote
Cultural adjustment and assimilation	Female, 63 years old	"When you come first time everything is very different than what we are used to back home. So, it definitely requires adjustment and a shift in the way you think about things”
	Male, 71 years old	"In India, because of the cultural setup and the neighbor setup, of course, it's changing with bigger cities there too becoming more metropolitan. But over here more so because the society is such that you may not even know who your neighbor is. That doesn't happen in India"
Social isolation, loneliness and the impact of immigration on social networks	Female, 59 years old	"In India, we were used to just drop in anybody's house … but here, all these numbers (of close friends) were almost in single digits, or even less"—Male, 71 years old
	Female, 59 years old	"Here, we have to make friends. So that feeling loneliness, It's more about the lifestyle here"
	Female, 59 years old	"It is very lonely at some points. Um, there was no one to call for help if I need any help. My children live in another city and I do not have family nearby"
	Male, 68 years old	"Back in India, we have so much support and so much social structure and you come here and there is nobody and nothing"
	Female, 79 years old	"Loneliness is one thing … that impacts your mental health. I think depression is much more in the Indian community than in other communities here, but we do not like to admit it to others"
	Female, 64 years old	"Especially when the kids left home … you're just constantly waiting for them to call or visit, so the loneliness hits again at that point"
Challenges in maintaining cultural	Male, 59 years old	"That was a challenging part, keeping the balance of both (Being Indian and American). I want them (my children) to be as Indian as they can, but at the same time, I don't want them to feel left out when they go out in this society”
	Female, 62 years old	"I feel that kids are at a place where at school they're exposed to American culture, but at home, we try to expose them to Indian culture, and they get confused as to what they should follow"
Resilience and adaptation	Female, 59 years old	"Learning is a part of your life and that keeps you alive. Without challenges, you'll be totally idle and that may lead to being depressed or unhappy when you move from one place to another"
	Male, 63 years old Female, 65 years old	“I always say be positive. That is my motto” “I kept myself so much busy that I did not have time to think about any negative. And I have always kept positive company around me”

### 4.1 Cultural Adjustment and Assimilation

Participants expressed that adjusting to a new cultural environment in the US posed significant challenges, particularly when it came to navigating social structures, cultural norms, and community interactions that differed greatly from those in their home country (India). Many highlighted how, upon arriving in the US, the cultural landscape felt unfamiliar, prompting a shift in mindset and expectations. They pointed out that the sense of community and connection they were accustomed to in India, where neighbors often formed close‐knit networks and interactions were more frequent and personal, was notably absent in the US. In contrast, participants described a more individualistic society where it was common not to know or interact with neighbors, which was a major adjustment. This cultural difference often left participants feeling isolated, as they had to adapt to a more impersonal social environment that lacked the communal bonds they had relied on in their home country. As a result, many faced emotional challenges as they worked to rebuild social connections in this new and unfamiliar context.

When you come first time everything is very different than what we are used to back home. So, it definitely requires adjustment and a shift in the way you think about things—Female, 63 years old

### 4.2 Social Isolation, Loneliness, and the Impact of Immigration on Social Networks

The transition to life in the US had a significant impact on the social networks of participants, many of whom struggled to build new friendships and maintain connections with old ones. Unlike in India, where socializing was more spontaneous and close‐knit networks were common, participants found that forming friendships in the US required more effort. They remarked on how the number of close friends dwindled, with many finding their social circles reduced to only a few individuals. This difficulty in forging new relationships was compounded by cultural and generational differences, which created barriers to forming the kind of deep, effortless connections they were accustomed to in their home country. Additionally, the lifestyle in the US, which many described as more individualistic, contributed to feelings of loneliness, as participants had to actively seek out friendships rather than rely on the organic, informal social interactions they once had. This shift made it challenging for them to recreate the same sense of community they had back home, leaving many feeling isolated in their new environment.

In India, we were used to just drop in anybody's house … but here, all these numbers (of close friends) were almost in single digits, or even less—Male, 71 years old

Many participants shared feelings of loneliness and social isolation, particularly after immigrating to the US. Participants’ accounts reflected both objective isolation, including smaller social networks and the absence of nearby family, and subjective loneliness, the felt experience of disconnection that persisted even when some social contacts remained. This sense of isolation was often intensified by the absence of a close‐knit community, family, and friends, which left them feeling disconnected. Participants described how they lacked the support systems they were used to in India, where strong social networks provided a sense of belonging and assistance. For example, some noted that there was no one nearby to turn to in times of need, with children living in different cities and extended family far away. The stark contrast between the rich social support systems in India and the more isolated environment in the US contributed to feelings of loneliness.

This emotional strain was further exacerbated as participants adjusted to life without the immediate presence of loved ones. Some mentioned how the departure of children from the household deepened their sense of isolation, as they found themselves waiting for occasional phone calls or visits. Others reflected on how loneliness took a toll on their mental health, with some acknowledging that depression might be more prevalent in their community but often goes unspoken due to cultural stigmas. These experiences highlight the emotional challenges many older Asian Indian immigrants face, compounded by the lack of familiar social structures and support in their new environment.

It is very lonely at some points. There was no one to call for help if I need any help. My children live in another city and I do not have family nearby—Female, 59 years old

### 4.3 Challenges in Maintaining Cultural Identity

Participants spoke about the difficulties of maintaining their cultural identity while navigating the process of assimilation into American society. A recurring theme was the struggle to balance preserving their cultural traditions with adapting to the cultural norms of the US, particularly when it came to raising their children. Many expressed a desire for their children to remain connected to their Indian heritage, but they also wanted to ensure their children felt accepted and integrated into American society. This balancing act often created challenges, as participants tried to instill Indian values and traditions at home, whereas their children were simultaneously exposed to American culture at school and in the broader community. Some participants noted that this dual exposure often left their children feeling conflicted about which culture to embrace, highlighting the complexity of maintaining cultural identity in a multicultural environment. These accounts reflect acculturative stress, as participants described the psychological strain of negotiating competing cultural expectations, working to preserve heritage values within the home while simultaneously supporting their families’ integration into US society.

That was a challenging part, keeping the balance of both (Being Indian and American). I want them (my children) to be as Indian as they can, but at the same time, I don't want them to feel left out when they go out in this society—Male, 59 years old

### 4.4 Resilience and Adaptation

Despite the challenges they faced, many participants displayed resilience and a positive outlook, finding ways to adapt to their new environment and maintain a sense of purpose. Several emphasized the importance of continuous learning and embracing challenges as a way to stay engaged and fulfilled, viewing adaptation as an opportunity for personal growth rather than a burden. Staying active and keeping busy was a common strategy, helping participants avoid dwelling on negative thoughts or feelings of isolation. Maintaining a positive mindset also played a crucial role, with some participants adopting mottos of optimism and surrounding themselves with supportive, uplifting company. These strategies allowed them to navigate the difficulties of immigration while fostering a sense of resilience and well‐being in their new surroundings.

I kept myself so much busy that I did not have time to think about any negative. And I have always kept positive company around me—Female, 65 years old

## 5 Discussion

Our study on older Asian Indian immigrants revealed several key themes related to their experiences related to social networks, isolation, and loneliness. The participants, primarily aged 55–74 years and long‐term US residents, faced significant challenges in cultural adjustment and assimilation upon immigrating to the US. The experiences of social isolation and loneliness among older Asian Indian immigrants in our study align with existing literature on immigrant populations [20, 21, 22]. They experienced a marked decrease in the size and quality of their social networks, leading to feelings of social isolation and loneliness [23]. This was exacerbated by the contrast between the communal culture they were accustomed to in India and the more individualistic society in the US. Participants also struggled to maintain their cultural identity while adapting to American society, particularly when raising children caught between two cultures [24]. Despite these challenges, many participants demonstrated resilience and adaptation, employing strategies such as continuous learning, staying active, and maintaining a positive outlook to navigate their new environment. These findings highlight the complex interplay of cultural, social, and personal factors that shape the experiences of older Asian Indian immigrants in the US.

Our findings reveal that older Asian Indian immigrants experience significant challenges in maintaining and rebuilding social networks after relocating to the US. Participants reported a marked decrease in the size and quality of their social circles, with many struggling to form new friendships due to cultural and lifestyle differences. The perception of the US society being more individualistic in nature, contrasting sharply with the communal culture in India, contributed to feelings of isolation and loneliness [23]. This social disconnection was further exacerbated by the physical distance from family members and the lack of spontaneous social interactions that were common in their home country. The absence of familiar support systems and the difficulty in recreating a sense of community in their new environment led to increased feelings of loneliness and social isolation [24]. These findings align with previous research on immigrant populations, which has shown that the disruption of pre‐existing social networks can lead to social isolation and negatively impact mental health [25]. Viewed through the lens of Social Network Theory, migration disrupted participants’ bonding ties, the close, emotionally supportive relationships with family and long‐standing friends that had anchored daily life in India, whereas new bridging ties to the broader US community proved difficult to establish in a social environment perceived as individualistic. This erosion of bonding ties, coupled with limited opportunities to form bridging and linking connections, helps explain why participants experienced both an objective reduction in the size of their networks (social isolation) and the subjective distress of feeling disconnected (loneliness), even after decades of residence in the US.

The struggle to maintain cultural identity while adapting to US society emerged as a significant theme in our study. Participants frequently discussed the delicate balance of preserving cultural traditions and values within their families, especially in the context of raising children who are often caught between two cultural frameworks. Many participants desired for their children to retain strong connections to their Indian heritage while also feeling accepted and integrated into American society. This balancing act often created internal conflicts, as participants attempted to instill Indian values at home, whereas their children were simultaneously exposed to American culture in schools and the broader community [21]. The challenge of maintaining cultural identity in a multicultural environment was particularly evident in the participants’ narratives about their children's experiences of feeling caught between two cultures [20]. These findings are consistent with previous research on acculturative stress among Asian Indian immigrants, which has highlighted the strong desire to carry on their belief system even after immigrating [26, 27, 28]. Interpreted through Acculturation Theory, participants’ efforts to balance Indian heritage with participation in US society reflect an integration strategy within Berry's framework, and the acculturative stress they described arose largely from the tensions inherent in pursuing integration, particularly within intergenerational family contexts, where parents and children acculturated at different rates. This dissonance between parents’ preference for cultural maintenance and their children's socialization into US norms constituted a salient and recurring source of acculturative stress in participants’ narratives.

Despite the considerable challenges highlighted above, participants exhibited remarkable resilience in adapting to life in the US and maintaining a positive outlook. Many actively engaged in strategies to stay connected and fulfilled, such as keeping themselves busy with activities, seeking uplifting company, and adopting an optimistic approach. These coping mechanisms reflect a strong motivation to adjust to new circumstances and to create meaningful lives despite the limitations posed by distance from extended family, shifts in social support, and changes in lifestyle [29]. This sense of resilience is rooted in an emphasis on lifelong learning and personal growth, which participants described as crucial for navigating the challenges of immigration. By focusing on purposeful activities, many were able to foster well‐being, combat loneliness, and build a renewed sense of community [30]. These findings highlight the adaptability of older Asian Indian immigrants and suggest that resilience plays a pivotal role in mitigating acculturative stress and supporting overall mental health [31]. Although the coping strategies most prominent in participants’ narratives were individual in nature, including optimism, keeping busy, and lifelong learning, resilience among older immigrants is also embedded in broader social and community resources. Prior research suggests that religious participation, cultural and ethnic community organizations, family support, and transnational ties to India can buffer stress and foster belonging among older Asian Indian immigrants [32, 33]. Notably, many of our participants were recruited through cultural and faith‐based organizations, and their accounts of seeking supportive social circles point to the protective role of these community structures. Future research should more systematically examine these collective and structural sources of resilience alongside individual coping strategies.

One of the strengths of our study is the focus on an understudied population: older Asian Indian immigrants, whose experiences with social isolation and acculturative stress in the US are often overlooked. The use of in‐depth interviews provided rich, nuanced data that allowed us to capture the complexities of participants’ lived experiences, adding depth to existing literature on immigrant health and well‐being. Additionally, the study location in North Texas, an area with a significant and growing Asian Indian population, offers insights that may be broadly relevant to similar urban centers across the US. However, there are limitations. First, the sample size is relatively small, limiting the generalizability of our findings to all older Asian Indian immigrants in the US. The use of convenience sampling, which relied on local cultural organizations and social networks, may introduce bias, as participants who are already connected within the community may differ from those who are more isolated. Second, our sample was highly educated, predominantly composed of naturalized US citizens, and had lived in the US for an average of nearly 35 years. The experiences of older Asian Indian immigrants with limited English proficiency, lower educational attainment, more recent immigration histories, or weaker community ties, who may experience more severe and qualitatively different forms of social isolation, are therefore not well captured in our data, and our findings should not be assumed to transfer to these groups. Third, although women comprised 72% of our sample, our analysis did not systematically examine gender differences; women's and men's experiences of social isolation, caregiving expectations, family roles, and cultural adaptation may differ in important ways [34], and future research should explicitly examine these gendered dimensions. Fourth, participants varied considerably in age at immigration, years lived in the US, and employment and marital status, but our sample size precluded systematic comparison across these subgroups. Larger studies should examine how differing immigration histories and life circumstances shape distinct pathways into, and out of, social isolation. Finally, member checking was not conducted, which may limit the credibility of our interpretations. Despite these limitations, this study offers valuable insights and lays the groundwork for future research into the social and mental health needs of aging Asian Indian immigrants in the US.

These findings have implications for public health practice. Social isolation among older immigrants is not only an individual experience but also a community‐level and structural issue shaped by migration, language, family dispersion, cultural expectations, and limited culturally responsive services. Public health programs serving older adults should partner with cultural associations, faith‐based organizations, senior centers, and primary care settings to identify socially isolated older Asian Indian immigrants and connect them to culturally meaningful opportunities for social participation. Interventions may include peer‐support groups, intergenerational programming, culturally adapted mental health education, community navigation, and outreach through trusted community organizations. Such approaches may reduce loneliness while supporting cultural identity, resilience, and healthy aging among immigrant communities.

## 6 Conclusion

Older Asian Indian immigrants in the US face multiple challenges, particularly concerning social isolation, acculturative stress, and the struggle to maintain cultural identity. This emphasizes the need for culturally tailored strategies to address social and mental health disparities in this understudied population, including fostering social connections and promoting mental well‐being. Future research should build on these insights to develop targeted interventions that enhance quality of life and support aging well in immigrant communities.

## Author Contributions

**Nolan Kline**: writing – original draft, writing – review and editing. **RoiSan Nhpang**: investigation, writing – original draft, formal analysis, data curation. **Stacey Griner**: writing – original draft, writing – review and editing. **Shilpa Patil**: investigation, writing – original draft, data curation. **Sarah Alkhatib**: writing – original draft, writing – review and editing. **Malinee Neelamegam**: conceptualization, investigation, funding acquisition, methodology, formal analysis, writing – original draft, writing – review and editing, project administration, supervision. **Anika Gupta**: writing – original draft, writing – review and editing.

## Ethics Statement

Ethical approval was obtained from the North Texas Institutional Review Board (Board Reference Number: 2022‐040). The principles of the Helsinki Declaration were followed.

## Conflicts of Interest

The authors declare no conflicts of interest.

## Data Availability

The data that support the findings of this study are available from the corresponding author upon reasonable request.
